# XitoSBML: A Modeling Tool for Creating Spatial Systems Biology Markup Language Models From Microscopic Images

**DOI:** 10.3389/fgene.2019.01027

**Published:** 2019-10-22

**Authors:** Kaito Ii, Kota Mashimo, Mitsunori Ozeki, Takahiro G. Yamada, Noriko Hiroi, Akira Funahashi

**Affiliations:** ^1^Systems Biology Laboratory, Department of Biosciences and Informatics, Keio University, Yokohama, Japan; ^2^Laboratory of Physical Chemistry for Life Science, Faculty of Pharmaceutical Sciences, Sanyo-Onoda City University, Sanyo-Onoda City, Japan

**Keywords:** spatial model simulation, spatial modeling, systems biology markup language, ImageJ, image processing

## Abstract

XitoSBML is a software tool designed to create an SBML (Systems Biology Markup Language) Level 3 Version 1 document from microscopic cellular images. It is implemented as an ImageJ plug-in and is designed to create spatial models that reflect the three-dimensional cellular geometry. With XitoSBML, users can perform spatial model simulations based on realistic cellular geometry by using SBML-supported software tools, including simulators such as Virtual Cell and Spatial Simulator. XitoSBML is open-source and is available at https://github.com/spatialsimulator/XitoSBML/. XitoSBML is confirmed to run on most 32/64-bit operating systems: Windows, MacOS, and Linux.

## Introduction

With the recent development of imaging technologies, we can quantitatively analyze spatial localization and concentration gradients of biochemicals within living cells (Chen et al., 2014; Keller and Ahrens, 2015). As a result, the importance of biochemical spatial localization and concentration gradients has become apparent. The effect of dynamics related to biochemical spatial distribution and cellular shape can be analyzed by using spatial model simulations (Rangamani et al., 2013).

However, in most spatial model simulations, cellular regions are defined as two- or three-dimensional spatial models based on simple mathematical equations. Because cell shape in such models differs from actual cells, these simulations will not produce appropriate results. Therefore, it is crucial to perform three-dimensional spatial model simulations by using spatial models with the actual cellular shape.

Moreover, the advance of microscopic imaging technologies has made it possible to acquire a considerable amount of microscopic cellular images from biological experiments. Therefore, providing a software tool that can automatically generate a spatial model from microscopic cellular images will play an essential role in Systems Biology.

Software tools such as Virtual Cell (Loew and Schaff, 2001), Smoldyn (Andrews et al., 2010), and Morpheus (Starruss et al., 2014) are capable of performing simulations with actual cellular shape. Virtual Cell is a computational environment for modeling and numerical simulation that provides a graphical user interface (GUI) to create biochemical network models and perform ordinary differential equations, partial differential equations, and stochastic numerical simulations. Virtual Cell is popular for its partial differential equation model simulation. Smoldyn is a stochastic model simulator that can perform spatial model simulation with a particle-based model. The molecules in the model are defined as particles that diffuse with Brownian motion. The software is mostly used for biochemical reaction simulation at the single-cell scale (e.g., nanometer-scale spatial resolution). Morpheus is a modeling environment for simulation with ordinary or partial differential equations and can be used to model a reaction-diffusion system for multiscale and multicellular systems. These software tools provide outstanding functionalities in terms of spatial modeling and simulation but are limited by their unique file format. Morpheus can import SBML (Systems Biology Markup Language) (Hucka et al., 2003) files but cannot import the spatial information from these files. Virtual Cell can import and export SBML files, including spatial information. Smoldyn supports the SBML file format using Virtual Cell as a proxy. Virtual Cell, Smoldyn, and Morpheus are also limited in that they do not offer an interface to overcome the difficulty of creating a spatial model from images.

Virtual Cell provides functionality to create a spatial model from microscopic images and export it as a spatial SBML document, but it only supports the import of grayscale or multichannel TIFF images; this is problematic for the following reasons: 1) When importing a grayscale image, users have to apply a segmentation task manually from the distribution of intensity provided by Virtual Cell. Because, in general, segmentation is a difficult task in image processing (Rajasekaran et al., 2016), manual segmentation for each organelle with the distribution of intensity would requires enormous modifications to each pixel in the image; and 2) Even though Virtual Cell supports the import of a multichannel TIFF image so that each channel can be segmented and assigned to each organelle, the program requires users to manually assign a membrane between two organelles in their model. The number of possible membrane positions would increase with *O*(*n* ^2^) [${(}_{2}^{n})$, where *n* is the number of organelles]. When the number of organelles is small, it would not be a critical problem for users but advances in microscopic technologies have enabled 9 to 24 multichannel images to be obtained for a single cell (Niehörster et al., 2016; Wei et al., 2017).

To solve these problems, we present XitoSBML, which is capable of creating spatial models from microscopic images in SBML format. XitoSBML uses images to construct spatial models with more flexibility in defining compartment shapes compared to models created with mathematical equations. Because XitoSBML is implemented as a plug-in for ImageJ (Schindelin et al., 2012; Schneider et al., 2012), users can call sophisticated segmentation algorithms for each channel through the user interface of ImageJ and directly apply the segmentation result to XitoSBML. This means that users can process images and create the spatial model within the same application. XitoSBML supports the import of plural segmented (binary) images for each organelle. Moreover, XitoSBML automatically assigns membranes between domains from given inclusion properties of organelles. XitoSBML also automatically adjusts the segmentation result so that users can create a spatial model without manually performing morphological operations and interpolation on the segmented images. SBML is compatible with more than 290 different software tools. Although only a limited number of software tools currently support spatial SBML simulation (Loew and Schaff 2001; Matsui et al., 2015), the demand for such modeling is increasing. We therefore expect that the number of spatial simulators will increase in the next few years. XitoSBML is a user-friendly and extensive modeling software, providing the environment to create a spatial model on the fly. Users may efficiently perform spatial model simulations and export the model to any compatible simulator.

## Materials And Methods

Here, we briefly describe XitoSBML and outline the process the program takes to create a model with JSBML 1.2 (Dräger et al., 2011). XitoSBML operates as a plug-in for ImageJ. Once the microscopic images are well organized and segmented (Nketia et al., 2017), XitoSBML can take them as input to create an SBML level 3 version 1 model with spatial processes (Schaff et al., 2015).

### Software Architecture

**Figure 1:**
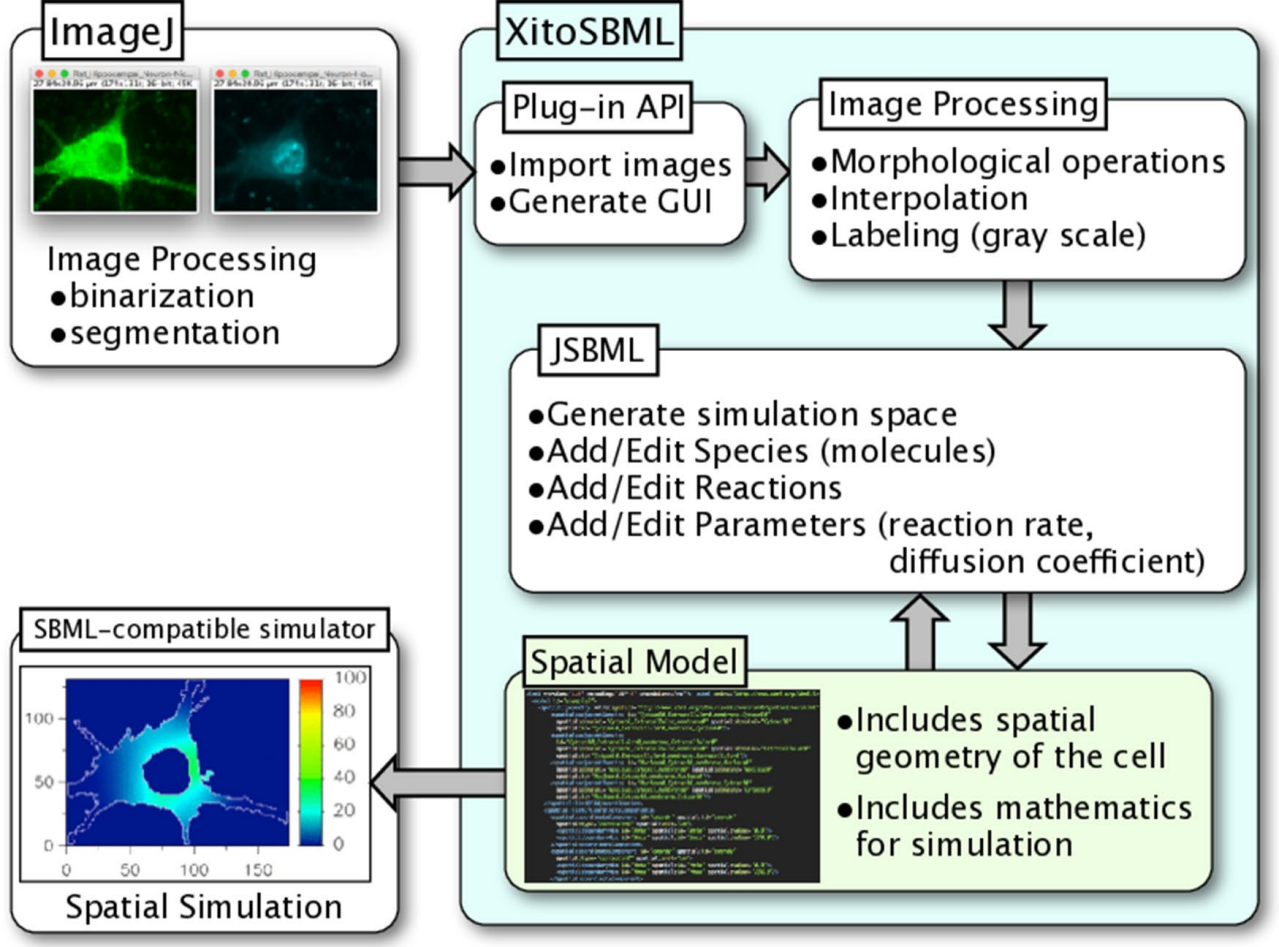
Architecture and workflow of XitoSBML. Inputs to XitoSBML are segmented images of a cell or spatial SBML model. The segmented images are easily generated by using existing functions in ImageJ. XitoSBML imports these images through ImageJ plug-in API and applies several image processing algorithms implemented in XitoSBML. The converted images are then exported to spatial SBML by using JSBML API. The generated spatial SBML model is used to perform a spatial simulation on SBML-supported simulators.

XitoSBML is open-source software distributed under Apache License, 2.0; it is written in Java and is platform-independent. XitoSBML uses an ImageJ plug-in application programming interface (API) to import images from ImageJ and to create the GUI (**Figure 1**). The imported images are passed to several image processing algorithms (morphological operations, interpolation, and labeling) implemented in XitoSBML. The JSBML API then converts the processed images to a spatial SBML model, which is converted to an SBML Level 3 Version 1 object that can be modified through the XitoSBML GUI. The converted spatial SBML model contains the spatial geometry of the original images as well as information on molecular concentrations, locations, biochemical reactions, and parameters. This information will be used by SBML-supported simulators to perform spatial simulation on the model.

#### Preprocess Of Images

XitoSBML takes in two-dimensional or z-stack three-dimensional images as input and creates a spatial model. Before doing so, the images must be in a specific format as outlined below. XitoSBML assumes the image is segmented and represented as a specific domain within the cell. Usually, the segmented image is a binary image that only contains black or white pixels. For example, input images with a segmented image of a nucleus and a cytosol will produce a spatial model with domains of extracellular matrix, cytosol, and nucleus. Therefore, to obtain a reasonable model, segmentation of the microscopic images is essential. ImageJ provides a variety of tools for this purpose. One of the benefits of XitoSBML is that one can process the image and create the model simultaneously on ImageJ: i.e., the user just has to process the images on ImageJ and import them into XitoSBML on ImageJ.

### Software Functionalities

#### Creating Spatial Model From Images

XitoSBML provides an easy-to-use GUI to create the spatial models. Before doing so, the microscopic images must be processed to binary representing one component of the cell; this can be performed on ImageJ. Given the input images, the software will generate a spatial model as follows.

**Figure 2:**
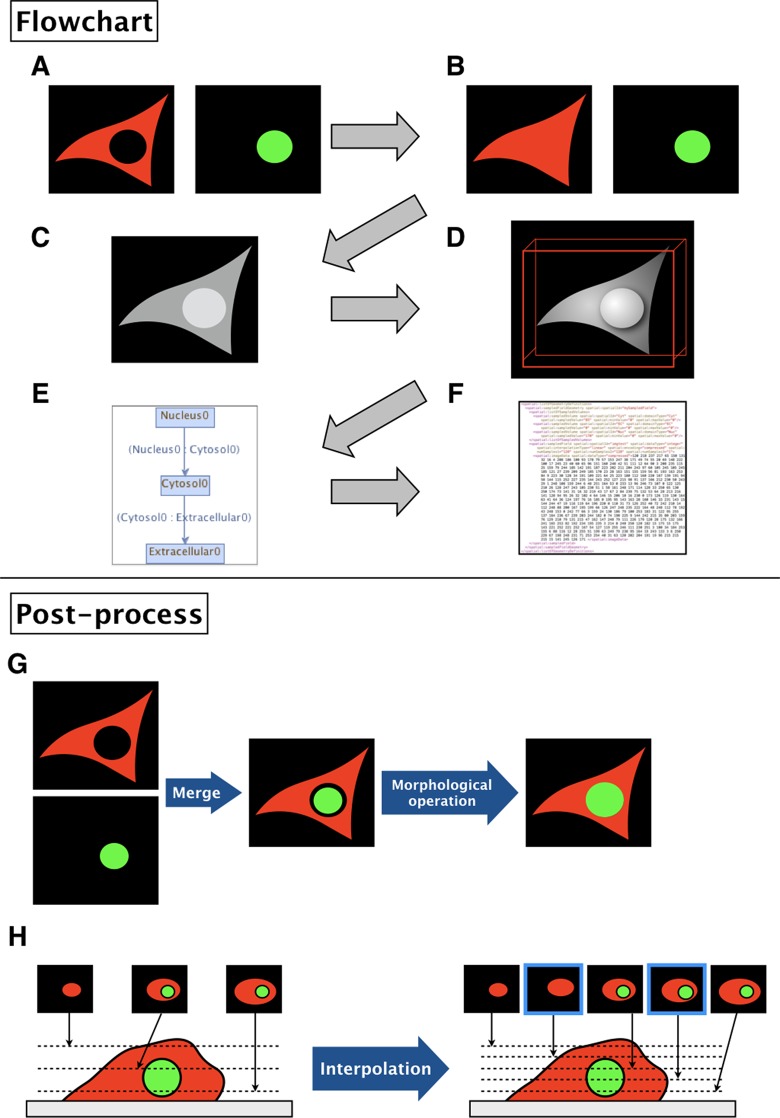
Flowchart of XitoSBML and post-processing of imported images in XitoSBML. **(A)** Segmented images of cytosol (red) and nucleus (green). The two images are in color for visualization purposes; however, when in actual use, they have to be binarized. These two images are set as inputs of XitoSBML. **(B)** Each input is filled by morphological operation and interpolated as necessary. The interpolation is performed with the nearest neighbor method. **(C)** The inputs are combined creating a new grayscale image. The domains, in this case, cytosol and nucleus, are assigned with the specific pixel value. If each domain overlaps with each other or creates a gap in between, the grayscale images are corrected. **(D)** The result of the grayscale image projected three-dimensionally with 3D viewer. Each color represents a different domain of the input. **(E)** Inclusion property within the model. The box refers to the domain, and the arrow refers to the adjacency of domains, which apparently corresponds with **(C)**. **(F)** The resulting model for the SBML document. **(G)** When a gap exists between two segmented regions (e.g., nucleus and cytosol), XitoSBML will automatically fill the gap by a morphological operation. **(H)** If the imported simulation space contains anisotropic voxels, XitoSBML will use the nearest neighbor method to interpolate z-slice images from the given input images.

1. The binary images (**Figure 2A**) are filled by morphological operation and interpolated if necessary for the sake of simulation (**Figure 2B**).
2. The software then combines the images into a single grayscale image, assigning a distinct pixel value to each component given by the input (**Figure 2C**).
3. After the software generates a simulation space from the given images, users may add molecular species, parameters, and reactions to the model.
4. The resulting image is visualized by surface rendering using a 3D Viewer (Schmid et al., 2010) (**Figure 2D**).
5. In addition, the inclusion property between domains is shown (**Figure 2E**). Using this relationship, one can check whether the domains in the model are biologically valid by showing which domains are adjacent to each other. Thus, the program can determine whether a model is biologically impossible: e.g., nucleus adjacent to the extracellular matrix.
6. Finally, the model is exported as an SBML document, along with the grayscale image (**Figure 2F**).

**Figures 2G**, H show the post-processing of imported images. When merging two segmented images, a gap might occur between two segmented regions (e.g., nucleus and cytosol) when the segmentation did not work correctly. XitoSBML will automatically fill the gap between these two regions by a morphological operation (**Figure 2G**). Most of the three-dimensional microscopic images (z-stack images) have low resolution on the z-axis. This induces anisotropic voxels in the spatial model, which in turn would cause inaccurate spatial simulation. To solve this problem, XitoSBML interpolates z-slice images from the given input images by the nearest neighbor method (**Figure 2H**). Common pitfalls of segmentation are covered by applying morphological operation and interpolation as a post-process.

**Figure 3:**
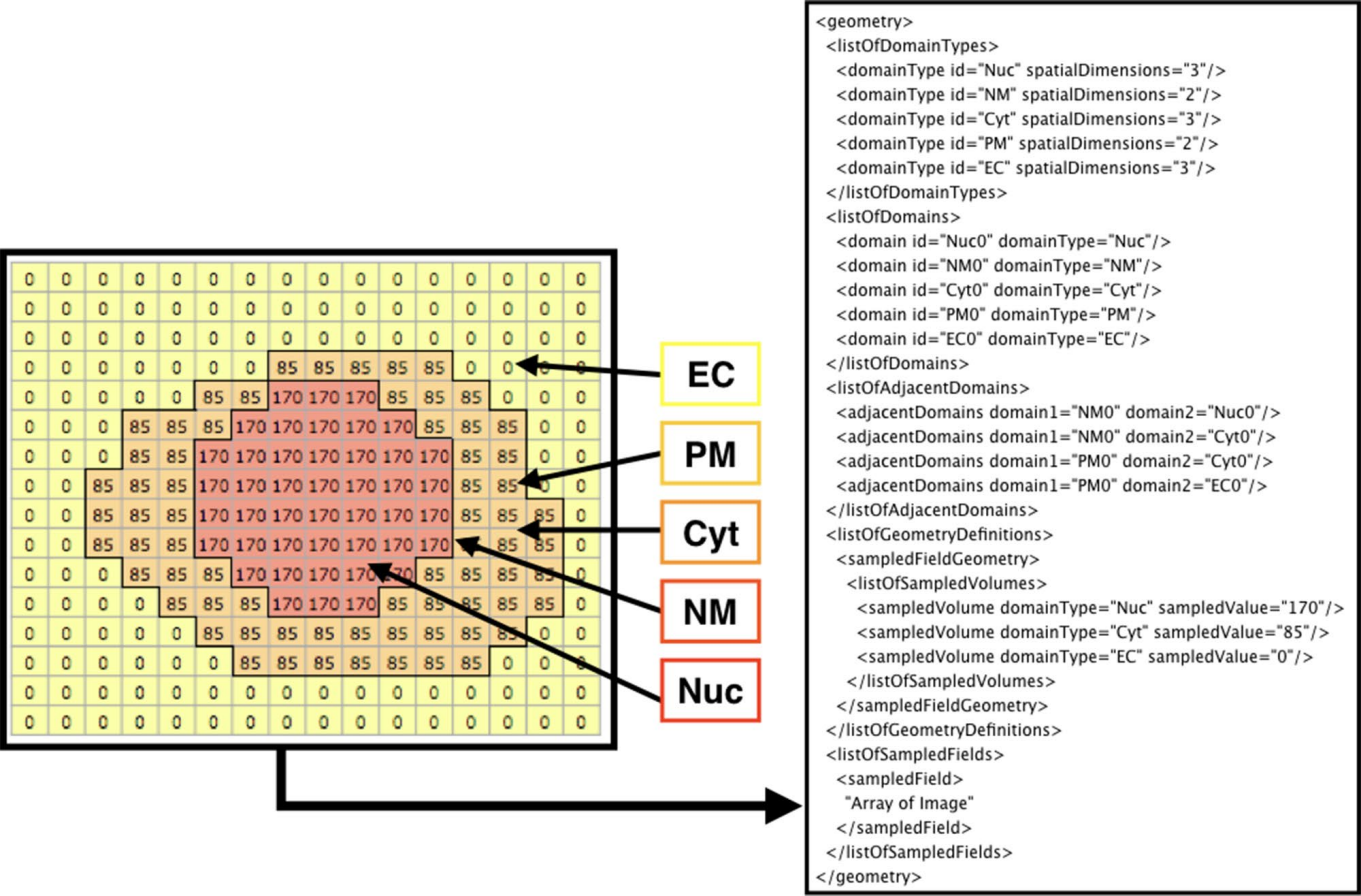
Diagram of how the image is written in the SBML document. The array on the left side is a sample image representing the grayscale image in **Figure 2C**. Each pixel value corresponds to a domainType, and every contiguous region on a domainType is defined as the domain of that domainType. Membranes are created between the domains and defined in the SBML document. In this instance, EC (extracellular matrix), PM (plasma membrane), Cyt (cytosol), NM (nuclear membrane), and Nuc (nucleus) are defined as domains. While creating the membrane, the adjacency of domains is resolved and defined as adjacentDomain. The corresponding value of the domain, excluding membranes, is defined as the sampledValue. Lastly, the array of the whole image is stored in the sampledField.

**Figure 3** shows how the domains are written in SBML. From the original image (e.g., **Figure 2A**), each domain is assigned a specific pixel value creating a single grayscale image (e.g., **Figure 2C**). In the example in **Figure 3** (left side), Nuc (nucleus) has a value of 170, Cyt (cytosol) has a value of 85, and EC (extracellular matrix) has a value of 0. From the grayscale image, the adjacency of domains is found, and membranes (with no thickness) are created between the domains. After the domains are created from given images, users can manually add molecular species and parameters (e.g., advection coefficient, boundary condition, or diffusion coefficient) into the necessary domains by XitoSBML (model editor). Then, all the information is written in an SBML document. While exporting the spatial model as an SBML Level 3 Version 1 document, XitoSBML executes both syntax and semantic validation on the SBML core package by using an API provided by JSBML and executes syntax validation on the SBML spatial package by using an online libSBML validator. Moreover, XitoSBML has a custom implementation of a validator that can semantically validate the spatial information inside the model.

The user needs to do only three easy steps to create a spatial model from images: 1) process the microscopic images to binary images, 2) add molecular species and parameters into the necessary domains, and 3) save the created model as a file.

#### Editing an Existing Model

XitoSBML also can handle existing spatial SBML models, thereby allowing users to modify their spatial SBML model by opening it from XitoSBML. Using the “run Model Editor” menu item from the “XitoSBML” plug-in menu, the molecular species and parameter in a model can be modified. With the correct version and extensions, any model can be modified.

## Results

**Figure 4:**
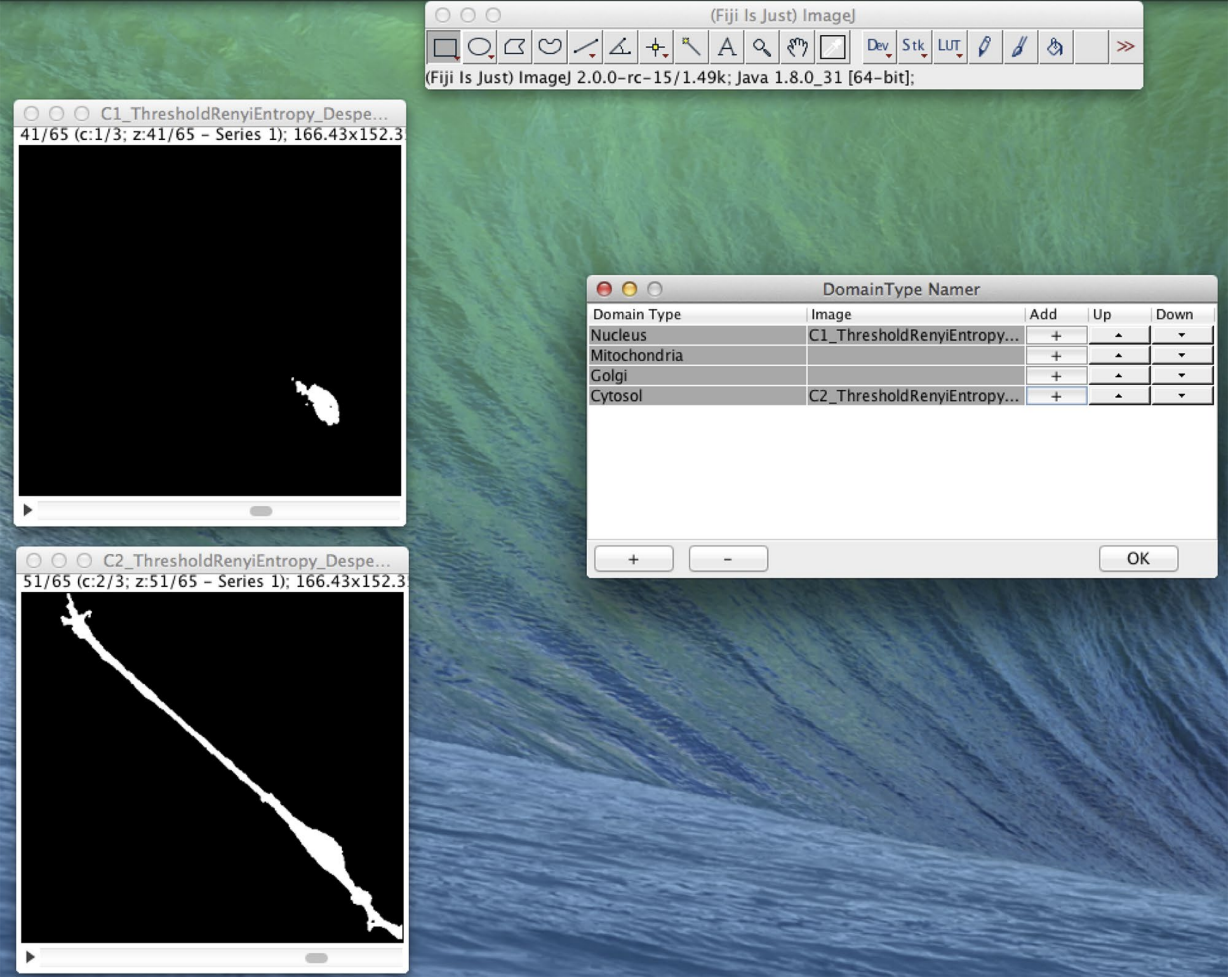
Screenshot of XitoSBML generating an SBML document from microscopic images. The segmented three-dimensional SH-SY5Y cell images are shown on the left. Top left is the nucleus, and bottom left is the cytosol. On the right is the interface of XitoSBML that allows the user to add the images to the corresponding domainType.

In **Figure 4**, we present an example of the use of XitoSBML software to demonstrate the basic work flow. As an input, we will use three-dimensional images of SH-SY5Y cells, which are derived from human neuroblastoma. Before construction of the model, the images were segmented using ImageJ, with each segmented image representing a geometry of a domain in a cell.

XitoSBML offers an easy way to create a spatial model from images. The obtained spatial model is usable for spatial model simulation with the appropriate simulator. Below, we present the result of a spatial model simulation with a spatial model exported from XitoSBML to validate the usefulness of the obtained spatial model. As an example simulator, we chose Spatial Simulator (Matsui et al., 2015), which is an in-house software implemented as a partial differential equation simulator specialized for SBML documents. Before performing the simulation, the XitoSBML output model requires further modification: information on biochemical reactions must be added because Spatial Simulator lacks the ability to add this information. XitoSBML provides a GUI for this purpose; users can add molecular species, reactions, parameters, and reaction rates to the model. In this example, the model is the simple transportation of molecules from Cyt to EC and simple diffusion combined. The result of the spatial model simulation is shown in **Figure S1**.

Even though the model is three-dimensional, to visualize the entire result, we show the results of a time series of a particular Z slice of the model. The colors inside the cell represent a concentration of molecule. By using XitoSBML and Spatial Simulator, users can easily create a spatial model, add mathematics to their model, and execute a spatial simulation from microscopic images.

## Discussion

Ever since the SBML Spatial Processes package was proposed, spatial models could be created in a standardized format. XitoSBML is one of the first software tools to create a pure SBML spatial model. Thus, we have provided a platform within a laboratory to perform spatial modeling in which acquisition of microscopic images and the addition of molecular species and parameters is conducted manually through the GUI of XitoSBML.

XitoSBML is a significant step toward more user-friendly tools for spatial biochemical modeling that provides the environment to create spatial models that reflect three-dimensional cellular geometry. It provides a GUI to easily create SBML Level 3 Version 1 documents and operates on ImageJ to simultaneously process images and create SBML documents. The exported model is compatible with SBML-supported software tools and can be used to perform spatial modeling. Thus, XitoSBML works as the gateway between bioimaging and spatial model simulation. As such, it provides a fast and easy way for biologists, who do not have detailed knowledge of modeling but can produce microscopic z-stack images, to perform spatial model simulations.

In the future, XitoSBML will be extended to automatically add the distribution of the initial concentration for each molecular species: in this new functionality, the fluorescent microscopic image of the localization of the molecule would be received and added as the distribution of initial concentration for that molecule in the SBML model.

## Data Availability Statement

The datasets generated and analyzed for this study can be found in https://github.com/spatialsimulator/XitoSBML/.

## Author Contributions

AF conceived and led the project. KI implemented the software and wrote the manuscript with TY. NH provided biological expertise. AF gave technical advice on the implementation. KM and MO provided advice on the image processing algorithms implemented in this software. All authors were involved in drafting or revising the content of the manuscript. All authors read and approved the manuscript.

## Funding

This work was supported by JSPS KAKENHI Grant-in-Aid for Scientific Research (B) (Grant Number 24300112) and the Imaging Science Project of the Center for Novel Science Initiatives (CNSI), National Institutes of Natural Sciences (NINS) (Grant Number IS271002).

## Conflict of Interest

The authors declare that the research was conducted in the absence of any commercial or financial relationships that could be construed as a potential conflict of interest.
