# Supplementary material for: XitoSBML: A Modeling Tool for Creating Spatial Systems Biology Markup Language Models From Microscopic Images
Source: Front Genet. 2019 Oct 22;10:1027. doi: 10.3389/fgene.2019.01027 (PMC6842926; doi:10.3389/fgene.2019.01027)
Supplement: Supplementary file 1 [file DataSheet_1.pdf]

Supplementary Material

1 SUPPLEMENTARY FIGURES

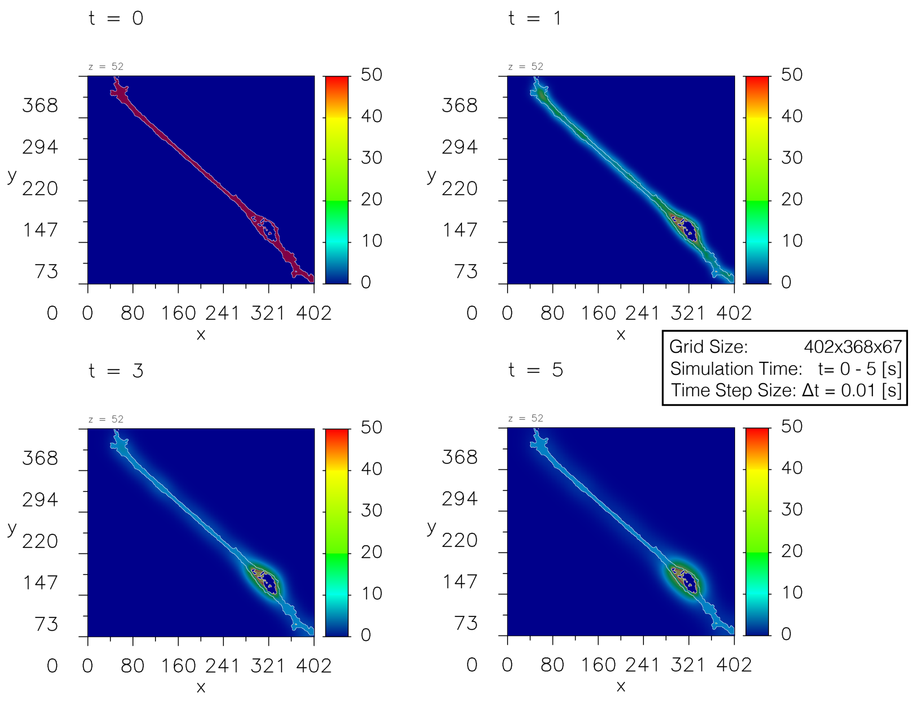

Figure S1. Simulation result created using Spatial Simulator using SH-SY5Y cell model as input.
